# Arrested Puberty in an Adolescent Male with Anorexia Nervosa Successfully Resumed with Multidisciplinary Care

**DOI:** 10.1155/2021/5512532

**Published:** 2021-07-13

**Authors:** Diana Simão Raimundo, Carolina Figueiredo, Ana Raposo, Bernardo Dias Pereira

**Affiliations:** ^1^Department of Pediatrics, Hospital Divino Espírito Santo de Ponta Delgada, Ponta Delgada, Portugal; ^2^Department of Endocrinology and Nutrition, Hospital Divino Espírito Santo de Ponta Delgada, Ponta Delgada, Portugal

## Abstract

The normal development of puberty depends on the specific pulsatility of gonadorelin, which is finely regulated by genetic and environmental factors. In the published literature, eating disorders figure as a cause of pubertal delay/arrest in females but are rarely considered in males with disordered puberty. A 16.7-year-old male was referred to the Department of Pediatrics with arrested puberty due to severe malnutrition in the context of food restriction. Past medical history was relevant for asthma. Generalized cachexia, facial lanugo hair, cutaneous xerosis, and Russell's sign were noted; he had a height of 155.5 cm (−2.5 SD; target height: 168 cm, −1.1 SD) and a BMI of 12.4 kg/m^2^ (−6.8 SD); left and right testicular volumes were 8 mL and 10 mL, respectively. He had a twin brother who had normal auxological/pubertal development (height: 167 cm, −1.05 SD; testicular volumes: 20 mL). Anorexia nervosa was diagnosed, and he was enrolled in a personalized treatment and surveillance program. “Nonthyroid illness” resembling secondary hypothyroidism was noted, as was low bone mineral density. Clinical and biochemical follow-up showed significant improvements in BMI (16.2 kg/m^2^, −2.55 SD), completion of puberty (testicular volumes: 25 mL), and reversion of main neuroendocrine abnormalities. Herein, we present an adolescent male with arrested puberty in the context of anorexia nervosa. The recognition of this rare condition in males allows a personalized approach to disordered puberty, with resumption of normal function of the hypothalamic-pituitary-gonadal axis and achievement of pubertal milestones.

## 1. Introduction

Puberty is the process of maturation of the hypothalamic-pituitary-gonadal (HPG) axis that culminates in the full achievement of final height, secondary sexual features, and fertility capacity [1]. This normal development depends on a specific pulsatility of gonadorelin (GnRH), finely regulated by genetic, hormonal, and environmental factors [1]. When pubertal development is absent or delayed, it may include normal variants or pathologic processes of the HPG axis, whereas if puberty is arrested, pathology is likely. The growth spurt may also be compromised in arrested puberty if not treated as the priming of the growth hormone (GH) axis by pubertal sex steroids is halted [2]. The most frequent cause of disordered puberty is constitutional delay of growth and puberty (53%), followed by functional hypogonadotropic hypogonadism (19%)—systemic diseases and psychological and nutritional causes—permanent hypergonadotropic hypogonadism (13%), and hypogonadotropic hypogonadism (12%) [3]. Among nutritional causes of disordered puberty, eating disorders should be considered; anorexia nervosa (AN) is an infrequent eating disorder with a lifetime prevalence of 0.6–2.2% [4, 5]. It is rare in adolescents (0.3%) [6], and as a cause of disordered puberty, it accounts for 0.9% of cases in one of the largest cross-sectional studies of adolescents with pubertal delay, where no males were reported [3]. In the Diagnostic and Statistical Manual of Mental Disorders (DSM) V criteria, AN is diagnosed if a person (1) restricts energy intake that leads to underweight, (2) has an intense fear of gaining weight or becoming overweight or has a persistent behaviour that prevents weight gain, despite being underweight, and (3) has a distorted perception of body weight and shape, has an inadequate influence of weight and shape on self-esteem, or denies the medical seriousness of underweight [7]. AN was classically a stereotyped diagnosis of females, clearly illustrated in the previous DSM IV criteria, which included amenorrhea as an obligatory feature to consider the diagnosis [8]. This entity is frequently overlooked in the differential diagnosis of an underweight male due to the rarity of the diagnosis in this gender, although males may represent a nonnegligible 25% of all AN cases [9]; noteworthy, males with AN have greater morbidity and mortality [10]. Due to very small sample sizes of adolescent males with AN in studies published to date [11], there is a paucity of data regarding the systemic impact of this eating disorder in this gender at younger ages, namely, in terms of neuroendocrinology [12]. We present a case of an adolescent male with arrested puberty due to severe undernutrition associated with AN. We also review the impact of AN and its treatment on the neuroendocrine axis, growth, and puberty.

## 2. Case Presentation

**Figure 1:**
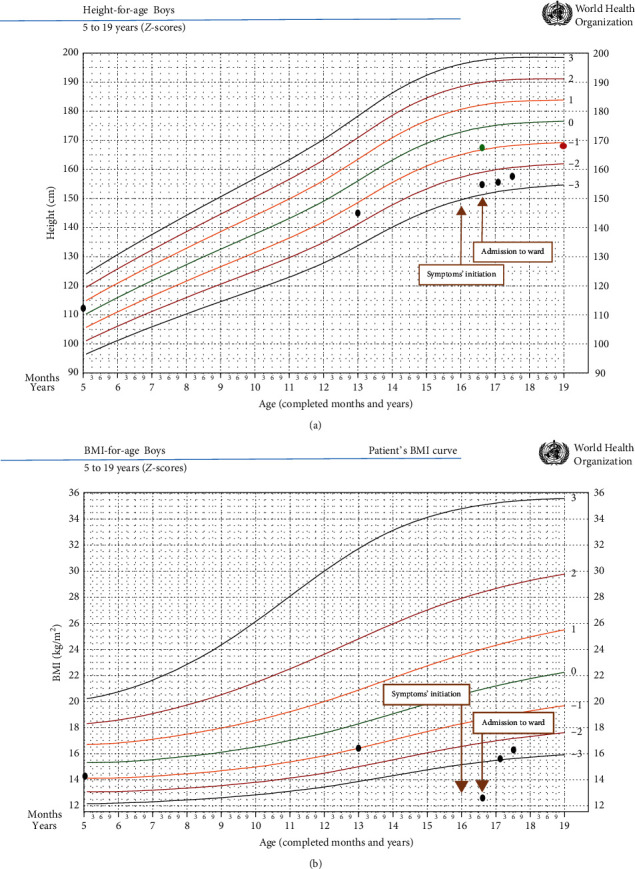
(a) Height chart (red dot: target height; green dot: twin's height; black dots: patient's height curve). (b) BMI chart (black dots: patient's BMI curve) of the patient showing shifting of *Z*-scores after an age of 13 years.

A 16.7-year-old Caucasian male was referred to Endocrinology due to nonfamilial short stature. He referred a weight loss of 13 kg in the last 6 months due to dietary restriction of carbohydrates and fat. He also referred decreased libido, asthenia, and decreased tolerance to physical efforts in the last month. He denied purgative behaviour and judged underweight as normal. He also denied any symptoms suggestive of an intracranial lesion. He had a personal history of asthma controlled with inhaled fluticasone and oral desloratadine. Family history was unremarkable. On physical examination, the patient had normal vital signs; generalized cachexia, facial lanugo hair, and cutaneous xerosis; Russell's sign was noted in his third left finger; height was 155.5 cm (−2.5 SD; target height: 168.9 cm, −1.1 SD), weight was 30.2 kg, and BMI was 12.5 kg/m^2^ (−6.8 SD); his height chart showed a progressive decrease in his linear growth, markedly (height velocity: 2.7 cm/year) after an age of 13 years (see Figure 1(a)); his BMI chart paralleled the height chart (see Figure 1(b)); the left and right testis measured 8 mL and 10 mL, respectively (Tanner stage: G3P3). He had a dizygotic twin with a height of 167 cm (−1.05 SD), normal BMI, and testicular volumes of 20 mL. A presumptive diagnosis of arrested puberty associated with an eating disorder was assumed, and the patient was referred to the Pediatrics Department, where he was admitted to the ward and enrolled in a multidisciplinary team approach, including Pediatrics, Psychiatry, Psychology, Endocrinology, and Nutrition. General biochemical evaluation is resumed in Table 1. An abdominal ultrasound excluded steatosis and other hepatobiliary diseases, the electrocardiogram was unremarkable, and chest radiography grossly excluded organic pathologies as causes of weight loss. Psychiatric evaluation suggested an atypical case of AN. A personality with perfectionist traits was revealed, encouraged by a central role played in his family. He also showed fusional relationship with his mother and lack of autonomy for his age. Unlike his twin, he received many family expectations regarding school performance and admission to a reputable university. Neither body image distortion nor anxiety disorder was perceived. The calorie counting was attributed to a need for control due to his perfectionist personality and obsessive personality traits. He was discharged with a weight of 34 kg, 14 days after initiation of a dietary plan and multidisciplinary support, which included family therapy. He was readmitted 3 months later due to a loss of 1.4 kg, and with further multidisciplinary support plus olanzapine 1.25 mg/day (maintained after discharge), his weight improved to 34.6 kg at discharge from the ward. After 6 months of follow-up, he had relatively unchanged height (156.5 cm, −2.5 SD) but improved in terms of BMI (15.6 kg/m^2^, −3.27 SD) and resumed puberty (testicular volumes: 12 mL). General biochemistry showed correction of initially abnormal parameters (Table 1); basal endocrine evaluation revealed normal gonadotropins and total testosterone for his evolving Tanner stage and “nonthyroid illness” resembling secondary hypothyroidism (Table 2). Dual-energy X-ray absorptiometry revealed low bone mineral density (BMD; *Z*-score lumbar spine: −3.4 SD; *Z*-score hip: −3.2 SD; lumbar spine BMD adjusted for height: −1.50 SD; hip BMD adjusted for height: −1.58 SD; reference/expected BMD adjusted for height: 0 SD), without imaging evidence of vertebral fractures. Bone age was estimated to be delayed by 8 months relative to chronological age. Currently, at 17.6 years old, he maintains his follow-up with a multidisciplinary team and his personalized nutritional plan, including daily supplementation with vitamin D (800 UI/day) and calcium (1200 mg/day). His height had a slight increase to 157.4 cm (−2.2 SD), as was his BMI to 16.2 kg/m^2^ (−2.55 SD). Testicular volumes increased to adult size (25 mL). Basal endocrine reevaluation showed increased total testosterone levels and correction of “nonthyroid illness”; high late-night salivary cortisol was noted (Table 2).

## 3. Discussion

Herein, we present a rare case of an adolescent male with arrested puberty due to severe undernutrition in the context of AN, which, with personalized treatment, could successfully resume his puberty. Data are lacking regarding the pubertal status (Tanner stage) of patients with AN, both at diagnosis and following treatment institution [11]. Available studies include mostly females and report the age of menarche as a marker of puberty. Premenarchal females with AN have invariably delayed menarche at diagnosis, but noteworthy, weight restoration allows its development in some but not all cases; males with AN have lower testicular volumes compared with age- and gender-matched controls, but pubertal growth spurt is usually able to be attained upon treatment initiation [11, 13–15]. In patients with AN, the LH pulse frequency resembles that of a prepubertal stage [16, 17]. The low levels of leptin that parallel the loss of adipose tissue in AN appear to be a crucial link between starvation and a disrupted reproductive system. Leptin acts on the hypothalamic arcuate nucleus upregulating the expression of kiss-1 and its protein (kisspeptin), a stimulator of GnRH neurons, and downregulating the expression of neuropeptide Y (a GnRH neuron inhibitor) [18]. Thus, hypoleptinemia is linked to an inhibitory drive to GnRH pulsatility. Ghrelin is a stomach-derived peptide that is elevated in AN; it stimulates appetite partially through neuropeptide Y activation, and this may be the link between increased levels of ghrelin and low levels of LH in females, as well as low levels of testosterone and testicular volumes in males with AN [13, 19]. The stressful mental milieu of patients with AN is linked to hyperactivity of the hypothalamic-pituitary-adrenal axis, which is driven by high levels of CRH [20]. Indeed, higher anxiety and depression scores obtained from women with AN correlate with serum cortisol [21]. Patients with AN maintain their circadian rhythm of cortisol, although at a higher set point [17, 21], and a significant proportion fails to suppress cortisol after low-dose dexamethasone tests [22]. Hypercortisolemia in patients with AN is linked to low GnRH drive and LH pulsatility, although the mechanisms behind this association are not fully clarified [23]. Normal levels of cortisol are usually achieved with weight recovery, although some patients remain hypercortisolemic despite an increase in BMI—as shown in our case—and other psychobiological factors may play a role in maintaining this overactive state [23]. Hypercortisolemia may also contribute to the biochemical pattern of “nonthyroid illness” that resembles central hypothyroidism [24], where starvation plays a major role in eliciting this dysfunction of the pituitary-thyroid axis [12, 17]. This abnormality is regarded as adaptive to spare energy for vital functions [15] but usually resumes with weight recovery [25], as shown in our case. Although our patient had a 33% increase in weight after 1 year of follow-up, he did not show catch-up growth. Studies that focused on the growth of paediatric patients with AN have shown no difference in final height between cases and age- and gender-matched controls, with catch-up growth seen on average at 1 year of follow-up [11]. However, some surveys have shown a failure to catch-up growth, even after weight restoration [11, 26]. The largest study that focused on the growth of young males with AN evidenced that catch-up growth was only attained in patients that had the diagnosis of AN before their pubertal growth spurt [26]. Additionally, although starvation and low BMI are sufficient to impede height and puberty to evolve, bone maturation is not delayed in proportion, and the epiphyseal growth plates continue to close [13, 26]. Considering that our patient had testicular volumes of 10 mL at diagnosis of AN and that pubertal growth spurt is expected to supervene from this Tanner stage onwards, we speculate that the failure to catch-up growth seen in our patient may have been related to a diagnosis of AN close to his pubertal growth spurt initiation, and the delay in recognizing and addressing AN until his 16 years old allowed a bone maturation that impeded the attainment of his target height. The growth failure seen in untreated patients with AN is also related to the disordered GH axis. GH levels are elevated in patients with AN due to low feedback control by IGF-1 and high levels of GH secretagogue ghrelin; IGF-1 is decreased due to hepatic GH resistance, evidenced by the low levels of its plasma binding protein, which constitutes the extracellular domain of the GH receptor [15, 19].

Malnutrition-induced hepatitis is common among individuals with AN [27, 28] as a likely explanation to elevations in aminotransferases seen in our patient. Less commonly, as part of the refeeding process, liver enzymes may also increase due to hepatic steatosis and can be distinguished from malnutrition-induced hepatitis by the finding of a fatty liver on ultrasonography [27]. Individuals with AN and malnutrition-induced hepatitis are also at an increased risk of hypoglycemia due to depleted glycogen stores and impaired hepatic gluconeogenesis [29]. Dyslipidemia is common in patients with AN, but its mechanisms remain unclear. A recent multicenter study showed evidence of elevated lipid concentrations in acutely ill patients with AN compared with healthy controls (HC), some of which persisted after partial weight restoration [30].

Our patient had low BMD. Adolescence is a period of increased bone accrual towards the attainment of peak bone mass, which is markedly impaired in adolescents with AN [13, 31], and earlier age at diagnosis of AN is related to higher severity of decreased BMD [31]. Consequently, individuals with AN at younger ages have a significantly higher risk of fractures (31–57%) when compared with age-matched controls and population-based incidence [31, 32]. Low BMD in AN is multifactorial: low testosterone in males impairs bone formation directly by reduced activation of osteoblasts (through the androgen receptor) and indirectly by low aromatization in adipose tissue to oestrogen, which acts on the osteoclast to reduce its mass (impairing responsiveness of osteoclast progenitor cells to RANKL and inducing apoptosis) and bone resorption [33]; low IGF-1 is also a critical determinant of BMD in patients with AN. Its levels directly correlate with bone turnover markers in adolescent males [13], and recombinant IGF-1 yielded significant improvements in BMD of patients with AN [15]; high cortisol levels are a strong predictor of low BMD in AN, and the well-known bone detrimental effects of hypercortisolemia may explain the lack of efficacy of oestrogen therapy for low BMD in patients with AN [21]; reduced lean mass directly correlates with BMD in males with AN [13], probably due to less stimulatory effects of muscular biomechanical forces on bone formation [13, 15]; BMI at diagnosis of AN directly correlates with the severity of BMD, and the magnitude of weight restoration is higher, so the best outcomes are achieved in terms of BMD [13, 15, 34]. There is a paucity of data regarding the benefits of pharmacologic options to increase BMD in adolescents with AN. The only small study in this age range included exclusively females and showed that alendronate increased BMD relative to placebo, although statistically nonsignificant; weight restoration was a significant predictor of bone mass accrual, emphasizing the importance of instituting a personalized treatment plan for patients with AN to restore weight and improve long-term bone health [34]. There are no published trials or recommendations to guide the treatment of hypogonadal adolescents with AN and osteoporosis (defined as BMD *Z*-scores <−2 SD and bone fractures) [15].

In conclusion, the presented case illustrates the importance of identifying the underlying aetiology of arrested puberty and a focused multidisciplinary approach to revert functional abnormalities of the HPG axis that allow pubertal resumption and completion. Early diagnosis and treatment are crucial to blunt the drivers of neuroendocrine abnormalities characteristics of AN, which may lead to suboptimal height gain, unachieved pubertal milestones, and a BMD prone to fractures.

## Figures and Tables

**Table 1:** General biochemical surveys since presentation to the last follow-up visit.

Parameter (units)	Follow-up period			Reference
	Presentation	6 months	15 months	
Hemoglobin (g/dL)	17.8	13.9	14.2	14–18
Hematocrit (%)	51.1	41.7	41.5	37–49
Creatinine (mg/dL)	0.87	—	0.79	0.7–1.3
AST (U/L)	378	34	24	<34
ALT (U/L)	532	39	23	10–49
GGT (U/L)	75	—	22	<73
Total/direct bilirubin (mg/dL)	0.66/0.16	—	0.32/0.13	0.3–1.2/<0.3
Albumin (g/dL)	4.1	—	—	3.5–5
INR	1.01	—	—	0.75–1.22
Glucose (mg/dL)	47	—	68	70–110
Total cholesterol (mg/dL)	235	—	—	<190
LDL cholesterol (mg/dL)	98	—	—	<130
Sodium (mmol/L)	144	—	143	135–145
Potassium (mmol/L)	4.07	—	4.6	3.5–5.5
Corrected calcium (mg/dL)	9.3	—	10	8.3–10.6
Phosphorus (mg/dL)	2	—	4.5	2.5–4.9
Magnesium (mg/dL)	2.2	—	—	1.6–2.4
25-Hydroxyvitamin D (ng/mL)	11.7	—	—	>30
Antitransglutaminase IgA (U/mL)	0.5	—	—	<7
Antitransglutaminase IgG (U/mL)	<0.5	—	—	—
Total IgA (mg/dL)	389	—	—	70–400
Erythrocyte sedimentation rate (mm)	2	—	—	<15

ALT: alanine transaminase; AST: aspartate transaminase; GGT: gamma-glutamyl transpeptidase; IgA: immunoglobulin A; IgG: immunoglobulin G; INR: international normalized ratio.

**Table 2:** Basal endocrine surveys from 6 months after presentation to the last follow-up visit.

Parameter (units)	Follow-up period		Reference
	6 months	15 months	
FSH (mUI/L)	3.4	3.4	1.5–12.4
LH (mUI/L)	4.11	4.8	1.7–8.6
Total testosterone (ng/dL)^‡^	317.7	651	249–836
TSH (UI/mL)	2.06	2.9	0.5–3.4
Free T4 (ng/dL)	0.79	1	0.85–1.37
Free T3 (ng/dL)	—	3	2–4.5
Prolactin (ng/mL)	10.6	—	4–15.2
IGF-1 (ug/L)^¥^	—	271	126–429
ACTH (ng/L)	—	23.1	7–63
Late-night salivary cortisol (nmol/L)^±^	—	14.4	<7

ACTH: adrenocorticotropic hormone; FSH: follicle-stimulating hormone; IGF-1: insulin-like growth factor-1; LH: luteinizing hormone; TSH: thyroid-stimulating hormone; T4: thyroxine; T3: triiodothyronine. ^‡^Collected at 08:30. ^¥^IGF-1 adjusted for age, in standard deviation (SD) score: −1.17 SD. ^±^Collected at 23:00.

## Data Availability

The data were obtained directly from the patient and his medical records.
